# Thoracic Myelopathy Caused by Ossification of the Yellow Ligament as a Distal Adjacent Segmental Disease after Posterior Cervical-Middle Thoracic Fusion Surgery

**DOI:** 10.1155/2020/7101496

**Published:** 2020-02-27

**Authors:** Toru Funayama, Kentaro Mataki, Tetsuya Abe, Hiroshi Noguchi, Kousei Miura, Hiroshi Kumagai, Katsuya Nagashima, Yosuke Shibao, Kosuke Sato, Masao Koda, Masashi Yamazaki

**Affiliations:** Department of Orthopaedic Surgery, Faculty of Medicine, University of Tsukuba, 1-1-1 Tennoudai, Tsukuba, 3058575 Ibaraki, Japan

## Abstract

Although adjacent segmental disease after posterior thoracic fusion surgery is rare, thoracic myelopathy due to ossification of the yellow ligament in the lower thoracic spine could develop because of mechanical stress when the lower instrumented vertebra has been set to the middle thoracic spine during the initial surgery. We report an extremely rare case of distal adjacent segmental disease after posterior cervical-middle thoracic fusion surgery requiring reoperation after exhibiting thoracic myelopathy due to ossification of the yellow ligament in the lower thoracic spine. An obese 53-year-old man with diabetes had undergone C3-6 laminoplasty and C7-T8 posterior decompression plus fusion due to ossification of the posterior longitudinal ligament at C5-T5. Although the short-term clinical course after the initial surgery was good, symptoms of myelopathy reappeared because of the ossification of the yellow ligament that developed at T9-11 with local flexibility. Thus, reoperation with fusion extension surgery was needed 1 year and 6 months after the initial surgery. Altogether, we recommend careful monitoring of the postoperative clinical progression and, if necessary, reoperation at the earliest.

## 1. Introduction

Adjacent segmental disease after posterior thoracic fusion surgery is much rarer compared to that after surgery involving the cervical or lumbar spine due to the reduced mobility of the thoracic spine [1]. Mechanical stress, however, is reportedly the most important cause of increased ossification of the yellow ligament (OYL) [2]. We report an extremely rare case of myelopathy caused by OYL in the lower thoracic spine as a distal adjacent segmental disease after posterior cervical-middle thoracic fusion surgery.

## 2. Case Presentation

**Figure 1:**
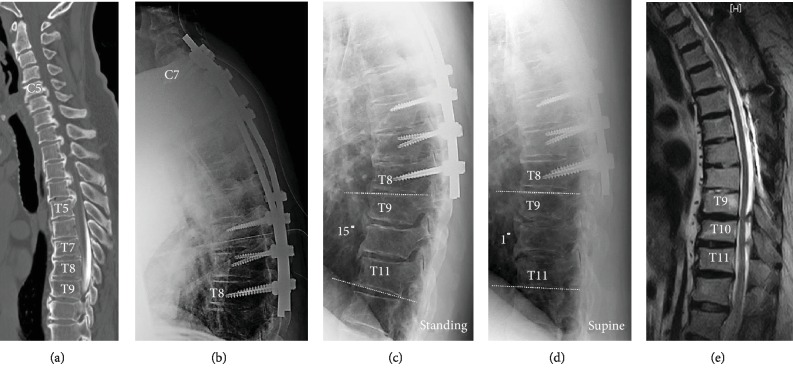
Spinal imaging before and after the initial surgery and before reoperation. (a) Ossification of the posterior longitudinal ligament was observed from C5 to T5. Bony fusion of the anterior longitudinal ligament was also observed at T7-9. (b) After initial surgery involving C3-6 laminoplasty and C7-T8 posterior decompression and fusion. (c, d) The kyphosis angle in T9-T11 was 15 degrees in the standing position (c) and 1 degree in the supine position (d) before reoperation. (e) MRI T2-weighted sagittal section. Spinal decompression in the initial surgical range was good. Advanced ossification of the yellow ligament at T9-11 and spinal cord compression at T9-10 are shown.

An obese diabetic 53-year-old man (body mass index, 36.3 kg/m^2^) presented to our hospital with severe myelopathy due to C5-T5 ossification of the posterior longitudinal ligament (OPLL) (Figure 1(a)). He had undergone C3-C6 laminoplasty and C7-T8 posterior decompression plus fusion a week after the first visit (Figure 1(b)). Although symptoms improved and the patient exhibited smooth gait after surgery, he experienced lower limb numbness and discomfort while walking 10 months postsurgically. The patient's spastic gait became more evident 1 year after surgery, and he needed Lofstrand crutches to walk.

The patellar and Achilles tendon reflexes were enhanced in both sides; however, manual muscle testing was normal. The patient experienced numbness in both lower limbs without urinary disorders. His Japanese Orthopaedic Association (JOA) score for thoracic myelopathy was 7 points out of 11 (full marks).

**Figure 2:**
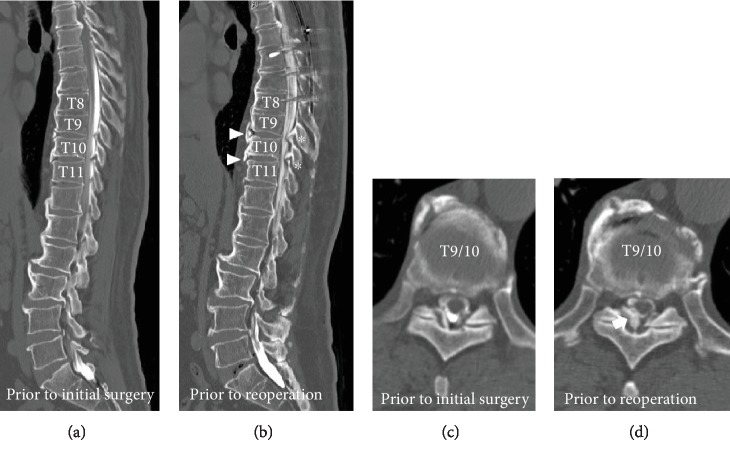
CT myelography images prior to initial surgery and reoperation. (a) Sagittal image prior to initial surgery. (b) Sagittal image prior to reoperation. Osteophytes (arrowheads) and markedly enlarged ossification of the yellow ligament lesion (asterisks) were observed at T9-11. (c) Horizontal image of T9-10 prior to initial surgery. (d) Horizontal image of T9-10 prior to reoperation. The ossification of the yellow ligament lesion is markedly enlarged (arrow), and spinal cord compression is apparent, which has shifted significantly to the left.

Imaging studies showed bone fusion in the posterior fusion surgery area, with no new spinal cord compression lesions. The lateral dynamic X-ray showed the kyphosis anglein T9-11 to be 15 degrees in the standing position (Figure 1(c)) and 1 degree in the supine position (Figure 1(d)), indicating local dynamic flexibility depending on the posture. MRI results showed advanced OYL at T9-11 and spinal cord compression at T9-10 (Figure 1(e)). When the pre- and postsurgical CT myelograms were compared, the sagittal images (Figures 2(a) and 2(b)) showed developed osteophytes and markedly enlarged OYL lesions were observed at T9-10 and T10-11. Furthermore, horizontal images (Figures 2(c) and 2(d)) showed that the OYL lesion at T9-10 was markedly larger and compressed the spinal cord. Since there was bony fusion of the anterior longitudinal ligament ossification at T7-9 before the initial surgery (Figures 1(a) and 2(a)), we diagnosed that myelopathy developed due to lower thoracic OYL as a distal adjacent disease.

**Figure 3:**
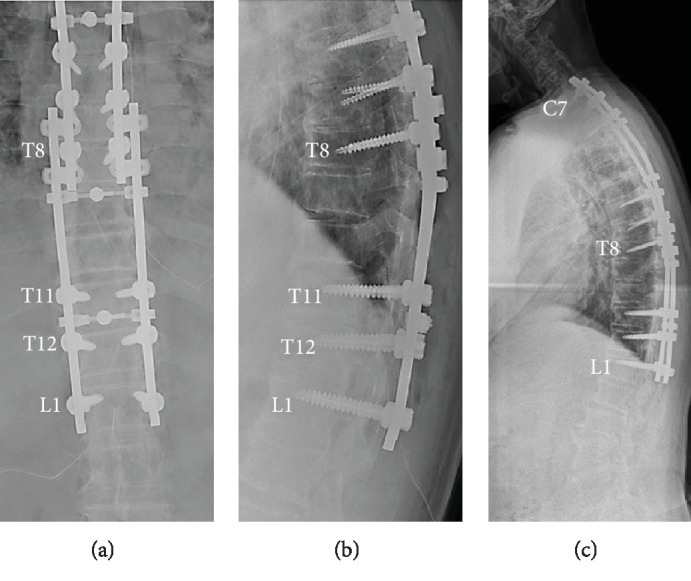
Images after reoperation. (a, b) Additional pedicle screws were inserted into T11, T12, and L1. Following laminectomy from T9 to T11, the rod was fastened and connected to the existing rod and domino connector to complete the posterior fusion extension surgery. (c) One year after reoperation, bone fusion and no loosening of the implant were observed.

The patient underwent reoperation 1 year and 6 months after the initial surgery. The posterior fusion extension surgery involved inserting pedicle screws into T11, T12, and L1, followed by laminectomy from T9-11 and resection of the OYL lesions (Figures 3(a) and 3(b)). The lesion at T9-10 showed dural ossification. Therefore, the entire ossified dura mater was carefully removed along the margins and repaired through patching with an artificial dura.

The symptoms of thoracic myelopathy gradually improved without complications following surgery, and the patient returned to work as a bus driver 6 months after reoperation. One year after reoperation, X-ray showed bone fusion and no loosening of the implant (Figure 3(c)). The patient displayed smooth gait without crutches, and the JOA score improved to 9.

## 3. Discussion

Long-range posterior decompression and fusion are often the first-choice surgical approaches for thoracic OPLL [3]. In this case, although OYL lesions were observed at T9-11 levels, there was no spinal compression at the same levels before the initial surgery. Therefore, we decided to consider T8 as the lower instrumented vertebra and to not perform prophylactic surgery on any lower vertebra. The short-term clinical course after initial surgery was good; thus, we believed that the initial fusion range was adequately selected.

Although myelopathy due to OYL in the lower thoracic spine as a proximal adjacent segmental disease after lumbar posterior fusion surgery has been reported [4], the same cannot be said for distal adjacent segmental disease.

Distal adjacent segmental disease is usually considered to occur at T8-9 when the lower instrumented vertebra is T8. However, in this case, bony fusion of the anterior longitudinal ligament was already observed at T7-9 before the initial surgery (Figures 1(a) and 2(a)). Therefore, the lower “fused” vertebra was T9 after the initial surgery. Apart from intervertebral degeneration due to mechanical stress [2], diabetes and obesity, which were seen in this case, can cause the development of OYL and can increase OYL [5]. Furthermore, the frequency of dural ossification increases with the severity of OYL [6]. According to the dynamic X-ray, which showed marked flexibility between T9 and T11 depending on the posture prior to reoperation (Figures 1(c) and 1(d)), we believed that there was high mechanical stress between vertebrae, extending caudally from the fusion range, especially at the T9-10 level. Thus, OYL had advanced to dural ossification within a short time period and ultimately led to thoracic myelopathy as a distal adjacent segmental disease.

Distal adjacent segmental disease can occur after posterior thoracic fusion surgery, and preexisting lower thoracic spine OYL can develop and present as thoracic myelopathy. Careful monitoring of the postoperative clinical progression and early reoperation, if necessary, are essential.
